# circ_0052184 Promotes Colorectal Cancer Progression via Targeting miR-604/HOXA9 Axis

**DOI:** 10.1155/2022/8583382

**Published:** 2022-08-27

**Authors:** Yandong Huang, Qinyang Bai, Zhanlong Wang, Hongbo Yu, Yanru Li, Hao Lu, Huimin Kang, Xuewei Shi, Kai Feng

**Affiliations:** ^1^Department of Tumor Center, The First Affiliated Hospital of Baotou Medical College, Inner Mongolia University of Science and Technology, Baotou, Inner Mongolia, China; ^2^Practical Education Skills Center of Baotou Medical College, Baotou Medical College, Baotou, Inner Mongolia, China

## Abstract

**Background:**

The mortality rate of colorectal cancer (CRC) ranks second. circRNAs are abnormal expression in some diseases, and their dysregulation is associated with cancer progression. Recent studies have shown that the malignant progression of colorectal cancer is inseparable from the abnormal expression of circRNAs.

**Methods:**

First, the circ_0052184 expression in clinical tissue and cell samples was analyzed by qRT-PCR. Then, we constructed circ_0052184-silenced CRC cells and detected by qRT-PCR. Furthermore, the proliferation ability of cells was detected by colony formation assay. Cell migration ability was tested by wound healing assay and transwell assay. Cell invasion ability was detected by transwell assay.

**Results:**

Expression of circ_0052184 was significantly increased in colorectal cancer cell lines and tissues. Silencing circ_0052184 affected the proliferation, migration, and invasion of colorectal cancer cells. miR-604 was targeted by circ_0052184. The downstream target of miR-604 was HOXA9, and silencing circ_0052184 inhibited HOXA9 expression. The existence of the circ_0052184/miR-604/HOXA9 regulatory network in colorectal cancer was validated. circ_0052184 promoted the occurrence and development of colorectal cancer by targeting the miR-604/HOXA9 axis.

**Conclusions:**

Our study revealed that the molecular mechanism of circ_0052184 regulated the miR-604/HOXA9 axis, which might promote the malignant progression of colorectal cancer cells.

## 1. Introduction

Colorectal cancer (CRC) has the second ranking mortality rate in the world and is the third ranking common cancer worldwide [1]. According to the National Comprehensive Cancer Network (NCCN) report, as of December 1, 2021, there were approximately 104,270 new cases of CRC worldwide and approximately 52,980 deaths annually [2]. The incidence of CRC may be affected by people's diet, weight, lifestyle, and other factors. However, the specific pathogenesis of CRC is complex. In recent years, the clinical treatment of colorectal cancer has made great progress. However, the survival rate of patients with advanced colorectal cancer within 5 years is still quite low. The most critical cause of death in CRC patients is the development and metastasis of CRC cells [3]. It is indispensable to further explore the mechanism of CRC progression to identify new targets.

Circular RNAs (circRNAs) are endogenous noncoding RNAs (ncRNAs) [4]. Covalently closed loops of circRNAs are formed by back-splicing, making this structure highly stable and conserved [5]. circRNAs can act as microRNA (miRNA) sponges to regulate the expression of key genes or function as transcriptional regulators [6]. Notably, tumor malignant progression has also been shown to be associated with the dysregulation of circRNAs [7]. However, the effect of circ_0052184 on CRC is unknown.

miRNA was paired with the 3′ end of target mRNA, degrading the target mRNA or inhibiting its translation into protein [8]. Many experiments have shown that the malignant development of human diseases is associated with miRNAs. Many miRNAs related to colorectal cancer have been discovered. For example, Yu et al. found that colorectal cancer migration and invasion were regulated by miR-17-5p [9]. Ding et al. found that apoptosis and cell cycle in colorectal cancer were regulated by miR-515-5p/MAD2L1 [10]. Kang et al. demonstrated that colorectal cancer development and patient prognosis were affected by miR-138 [11]. These studies suggested the impact of miRNAs on colorectal cancer. Existing studies have shown that miR-604 may regulate the occurrence and development of cancers such as gastric cancer and hepatocellular carcinoma [12]. However, few studies have explored the role of miR-604 in the malignant development of CRC.

Studies have found that there are many mechanisms of circRNAs. Among them, circRNAs are the most common as competing endogenous RNAs. At this time, the circRNA sponge absorbs miRNAs, thereby regulating the downstream target genes of miRNAs [13]. Interestingly, we found that circ_0052184 had the most elevated expression in colorectal cancer, and circ_0052184 was a molecular sponge of miR-604. HOXA9 is a member of the homeobox (HOX) family of highly conserved transcription factors that regulates the development of individual embryos [14]. Aberrant expression of HOX family proteins promotes the malignant progression of tumors. It has been reported that HOXA9 was regulated by miRNA targeting, thereby promoting the malignant development of tumors [15]. We experimentally confirmed the targeting relationship between miR-604 and HOXA9, which was regulated by circ_0052184. In this study, we found that circ_0052184 promoted colorectal cancer development by regulating the miR-604/HOXA9 axis.

## 2. Materials and Methods

### 2.1. Clinical Sample Collection

The colorectal cancer patients in our hospital were recruited as the research subjects, and the case detection results of the research subjects were all colorectal cancer. Cancer tissue and paracancerous tissue were collected during surgery. After sampling, the samples were quickly frozen in liquid nitrogen and stored in a -80°C refrigerator for subsequent operations. All subjects in the study had not received other radiotherapy and chemotherapy before the experiment. This study was approved by the ethics committee of our hospital, and all participating patients signed the informed consent. All experimental manipulations involved were performed in compliance with the *Declaration of Helsinki*.

### 2.2. Cell Culture

All cell lines were purchased from the BFB Cell Culture Bank (Shanghai, China). The cell lines involved the following: (1) NCM460 (normal colorectal cell line); (2) LoVo, HT29, HTC116, and SW480 (colorectal cancer cell line). These cell lines were cultured in DMEM (Waltham, MA, USA). The medium contained fetal bovine serum (10%), 100 U/mL penicillin, and 100 *μ*g/mL streptomycin (Thermo Fisher Scientific, MA, USA). Constant temperature incubators were used to cultivate the cell lines. Conditions were as follows: 37°C and 5% CO_2_. Subsequent experiments were mainly performed on cells in the logarithmic growth phase.

### 2.3. Bioinformatic Analysis

The online bioinformatics analysis website circinteractome was used to analyze the binding sites between circ_0052184 and miRNA. The possible binding sites between miR-604 and HOXA9 were analyzed by the bioinformatics analysis website starBase (https://starbase.sysu.edu.cn/).

### 2.4. qRT-PCR

TRIzol (Invitrogen, California, US) was used to extract RNA from CRC tissues and cells. Then SuperScript IV reverse transcription kit (Thermo Fisher Scientific) was used to synthesize cDNA. LabScript Two-Steps SYBR Green qRT-PCR Kit was used for qRT-PCR detection. Primer sequences are detailed in Table 1.

GAPDH or U6 were used as controls to normalize the relative expression levels.

Reaction conditions are as follows: predenaturation at 94°C for 5 min, 40 cycles of 94°C for 30 s, 55°C for 30 s, and 72°C for 30 s.

Relative expression levels of genes were calculated by 2^-△△CT^ method.

### 2.5. Transwell Assay

The successfully transfected LoVo and HT29 cell lines were collected. These cells were suspended in a serum-free medium. Cell suspension was added to the upper chamber of the transwell. The lower chamber of the transwell was medium supplemented with 20% fetal bovine serum. The cell compartment was removed from the incubator after 24 hours of culture. 4% paraformaldehyde (Beyotime Biotechnology, Shanghai, China) fixed the migrated cells. After fixation, cells were stained with 0.1% crystal violet (Beyotime Biotechnology) and photographed for counting after drying. In the invasion experiment, a layer of Millipore Matrigel (MA, USA) was coated on the surface of the transwell chamber. Other steps were similar to the migration experiment steps.

### 2.6. Colony Formation Assay

The successfully transfected LoVo and HT29 cell lines were seeded into 6-well plates, and the medium was changed every 3 days. After 14 days of culture, the previous medium was removed, and cells were fixed with 4% paraformaldehyde and then stained with 0.1% crystal violet. The colonies were photographed and counted under a microscope, and the colony formation rate was calculated.

### 2.7. Dual-Luciferase Reporter Assay

The wild-type and mutant circ_0052184 and HOXA9 luciferase reporter vectors were constructed. After the colorectal cancer cell lines LoVo and HT29 were seeded in 96-well plates for a period of time, the Wt or Mut luciferase reporter plasmids and miR-604 mimic or NC were cotransfected into LoVo and HT29 cells by Lipofectamine TM 2000 (Invitrogen). Cells were cultured for 48 h. The cellular luciferase activity of the different groups was detected by a Renilla-Firefly Luciferase Dual Assay Kit (MCE, Shanghai, China).

### 2.8. RIP

Targeting relationships between genes were examined using the Magna RIP™ RNA-Binding Protein Immunoprecipitation Kit (Millipore, Massachusetts, US). After the transfected were lysed with RIP lysis buffer, anti-Ago2 antibody or negative control IgG-conjugated magnetic beads were incubated with the RIP-supplemented buffer. Next, proteinase K was added to the mixture, and the mixture was incubated. The targeting relationships between genes in purified RNA was verified by qRT-PCR.

### 2.9. Wound-Healing Assay

The successfully transfected colorectal cancer cell lines were diluted and placed in 6-well plates for 24 hours and then streaked with a sterilized pipette tip. Photographs were taken at 24 h and 48 h to record the wound closure. Relative cell migration ratio = (0 h scratch area − 48 h scratch area)/0 h scratch area.

### 2.10. Western Blotting Assay

A RIPA lysis buffer (Beyotime, Shanghai, China) was used to extract total protein from tissues and cell lines. SDS-PAGE and polyvinylidene fluoride (PVDF) separate target proteins. Finally, the ECL kit (Beyotime) was used to detect total protein.

### 2.11. Statistical Analysis

All data in this experiment were analyzed with GraphPad Prism 8.0 (CA, USA). All experiments were performed at least three times. Data were expressed as mean ± standard deviation (*X* ± SD) Two groups were compared by *t*-test or one-way ANOVA. Pearson correlation analysis was performed to analyze the correlation between genes. The difference was statistically significant with *p* < 0.05.

## 3. Results

### 3.1. circ_0052184 Expression in CRC Tissues and Cells

**Figure 1:**
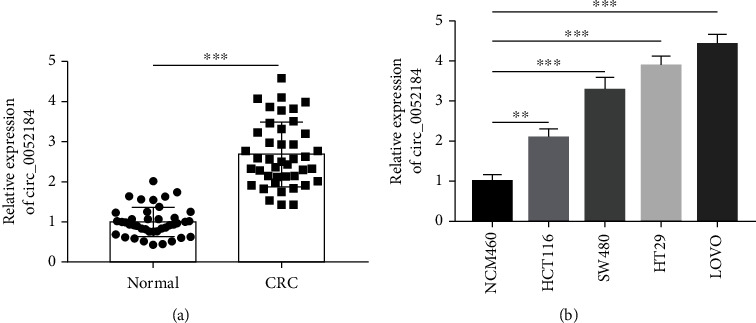
circ_0052184 was upregulated in colorectal cancer tissues and cell lines. (a, b) The expressions of circ_0052184 in CRC tissues (*n* = 42) (a) and cell lines (b) were detected by qRT-PCR. ^∗^*p* < 0.05, ^∗∗^*p* < 0.01, ^∗∗∗^*p* < 0.001.

First, we revealed the important role of circ_0052184 in the occurrence and development of CRC cells. The expression of circ_0052184 in cancer tissues and corresponding adjacent tissues of 42 CRC patients was analyzed by qRT-PCR. The patient details are shown in Table 2. The expression of circ_0052184 was increased in colorectal cancer tissues and cell lines (Figures 1(a) and 1(b)). We selected colorectal cancer cell lines (LoVo and HT29) with significantly increased expression of circ_0052184 for subsequent cell experiments.

### 3.2. Downregulation of circ_0052184 Affects Colorectal Cancer Cell Proliferation, Migration, and Invasion

**Figure 2:**
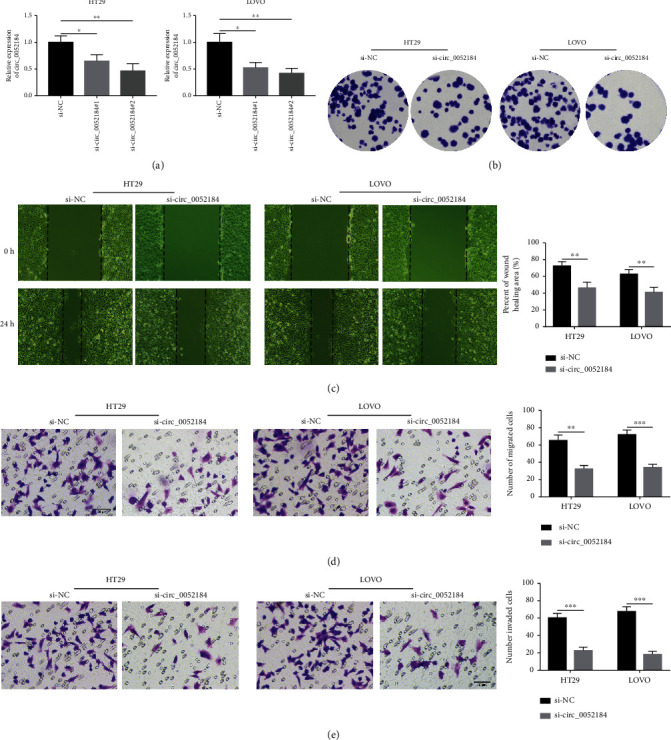
circ_0052184 silencing inhibited colorectal cancer cell proliferation, migration, and invasion. (a) The expression of circ_0052184 in LoVo and HT29 cells after transfection were detected by qRT-PCR. (b) Cell proliferation was detected by colony formation assay. (c, d) Cell migration was measured by wound healing assay (c) and transwell assay (d). (e) Cell invasion was detected by transwell assay. ^∗^*p* < 0.05, ^∗∗^*p* < 0.01, ^∗∗∗^*p* < 0.001.

circ_0052184 was silenced in LoVo and HT29. Then, the transfection efficiency was detected by qRT-PCR. It was shown that the expression of circ_0052184 was significantly reduced in the transfected cell line, which might be used for subsequent experiments (Figure 2(a)). Colony formation assays showed that LoVo and HT29 cell line proliferation abilities were inhibited after circ_0052184 was silenced (Figure 2(b)). Wound healing experiments and transwell experiments showed that after silencing circ_0052184, the migration ability of LoVo and HT29 cell lines was reduced (Figures 2(c) and 2(d)). In addition, transwell assays revealed that the invasion ability of LoVo and HT29 cell lines was inhibited after silencing of circ_0052184 (Figure 2(e)). Therefore, the above experiments indicated that silencing of circ_0052184 inhibited the malignant development of colorectal cancer cells.

### 3.3. circ_0052184 Targets miR-604

**Figure 3:**
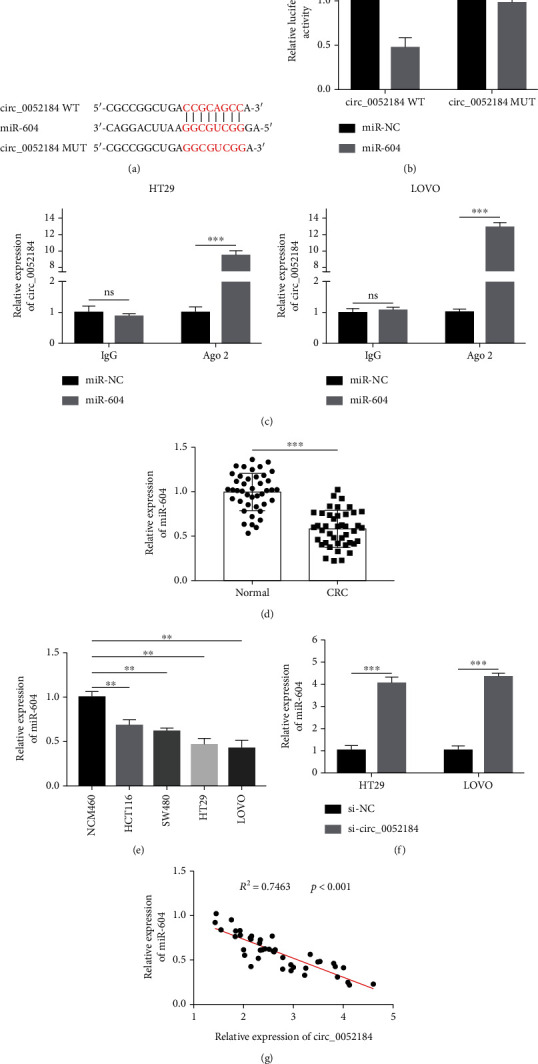
miR-604 is a downstream target of circ_0052184. (a) Bioinformatics was used to predict the complementary sequence between miR-604 and circ_0052184 by circinteractome. (b, c) Dual-luciferase reporter assay (b) and RIP assay (c) were used to verify the binding relationship between miR-604 and circ_0052184. (d, e) qRT-PCR was used to detect the expression of miR-604 in CRC tissues and cell lines. (f) qRT-PCR was used to measure miR-604 expression in LoVo and HT29 after circ_0052184 inhibition. (g) Pearson correlation between miR-604 and circ_0052184 in CRC tissues. ^∗^*p* < 0.05, ^∗∗^*p* < 0.01, ^∗∗∗^*p* < 0.001.

To further explore the mechanism of circ_0052184 regulating the development of colorectal cancer cells, the target genes of circ_0052184 were predicted through the online bioinformatics analysis website circinteractome. The results showed that there was a binding site between circ_0052184 and miR-604 (Figure 3(a)). The dual-luciferase reporter gene assay and RIP assay further confirmed the targeting relationship between circ_0052184 and miR-604. In the dual-luciferase reporter assay, overexpression of miR-604 significantly reduced the luciferase activity of circ_0052184-WT but had no effect on the luciferase activity of circ_0052184-MUT (Figure 3(b)). Meanwhile, RIP experiment showed circ_0052184 competitively binding to miR-604 in the RNA-Ago2 antibody (Figure 3(c)). The results showed that the expression of miR-604 was lower in colorectal cancer tissues compared with paracancerous tissues (Figure 3(d)). The expression of circ_0052184 in colorectal cancer cell lines was lower than that in human normal colorectal cancer cell lines (Figure 3(e)). When the expression of circ_0052184 in LoVo and HT29 cell lines was inhibited, the expression of miR-604 was increased (Figure 3(f)). Pearson correlation analysis clarified that circ_0052184 expressions and miR-604 expressions were negatively correlated (Figure 3(g)). In conclusion, circ_0052184 regulated colorectal cancer development by inhibiting the expression of miR-604.

### 3.4. circ_0052184 Regulates CRC Cell Development by Targeting miR-604

**Figure 4:**
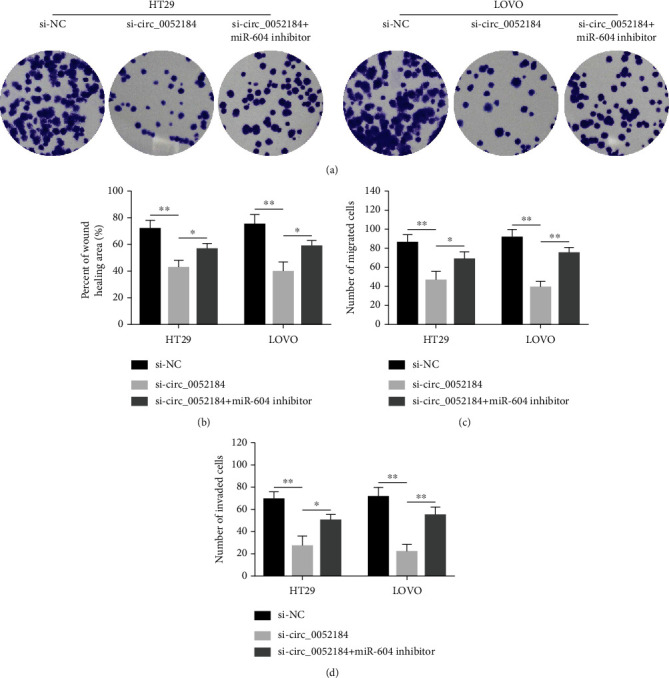
circ_0052184 regulated colorectal cancer cell proliferation, migration, and invasion by targeting miR-604. miR-604 inhibitor and siRNA-circ_0052184 or corresponding control were transfected into LoVo and HT29 cells. (a–d) Cell proliferation, migration, and invasion of cells were detected by colony formation assay (a), wound healing assay (b), and transwell assay (c, d). ^∗^*p* < 0.05, ^∗∗^*p* < 0.01, ^∗∗∗^*p* < 0.001.

A series of in vitro experiments were conducted to explore the regulatory effect of circ_0052184 on miR-604 in CRC cells. The results of colony formation assays showed that silencing the expression of circ_0052184 inhibited the proliferation of colorectal cancer cells, while inhibiting the expression of miR-604 reversed the inhibitory effect of circ_0052184 on cell proliferation (Figure 4(a)). Next, the results of the wound-healing assay and transwell assay showed that silencing the expression of circ_0052184 inhibited the migration of CRC cells, while inhibiting the expression of miR-604 reversed the migration of CRC cells (Figures 4(b) and 4(c)). Finally, transwell assays showed that when the circ_0052184 expression was silenced, it inhibited CRC cell invasion. However, inhibiting the expression of miR-604 reversed the CRC cells invasion (Figure 4(d)). From the above results, it concluded that silencing circ_0052184 inhibited CRC malignant progression, and inhibiting the expression of miR-604 reversed this process. In short, the circ_0052184/miR-604 axis regulated CRC cell development.

### 3.5. circ_0052184 Regulates HOXA9 Expression by Targeting miR-604

**Figure 5:**
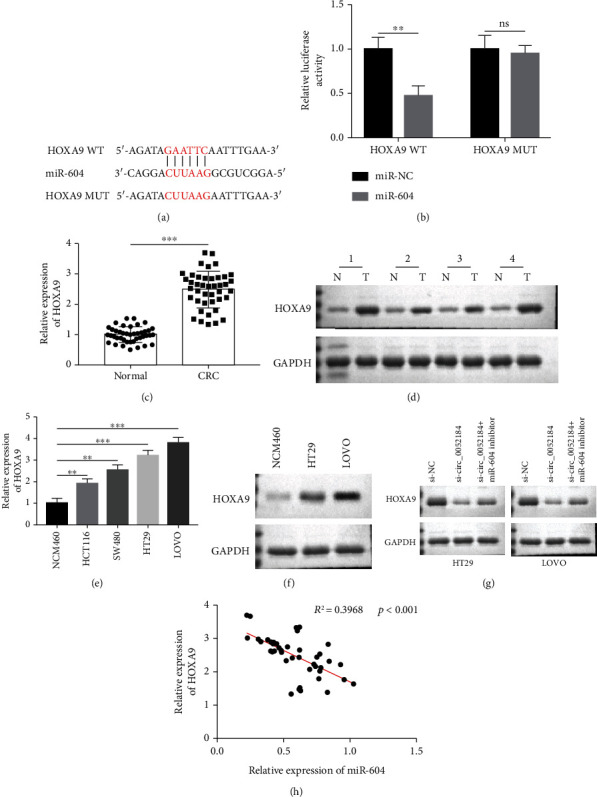
circ_0052184 regulated HOXA9 expression by targeting miR-604. (a) starBase was used to predicted the complementary sequence between miR-604 and HOXA9 3′UTR. (b) Dual-luciferase reporter gene assay was used to confirm the binding relationship between miR-604 and HOXA9. (c, d) HOXA9 mRNA and protein level were measured by qRT-PCR (c) and Western blot (d) in CRC tissues. (e, f) HOXA9 mRNA and protein levels were measured by qRT-PCR (e) and Western blot (f) in CRC cell lines. (g) The protein expression of HOXA9 was measured by Western blot in CRC cells transfected with control siRNA (si-NC), circ_0052184 siRNA (si-circ_0052184), or cotreated with si-circ_0052184 and inhibitors of miR-604, respectively. (h) Pearson correlation between HOXA9 mRNA expression and miR-604 expression in CRC tissues. ^∗^*p* < 0.05, ^∗∗^*p* < 0.01, ^∗∗∗^*p* < 0.001.

**Figure 6:**
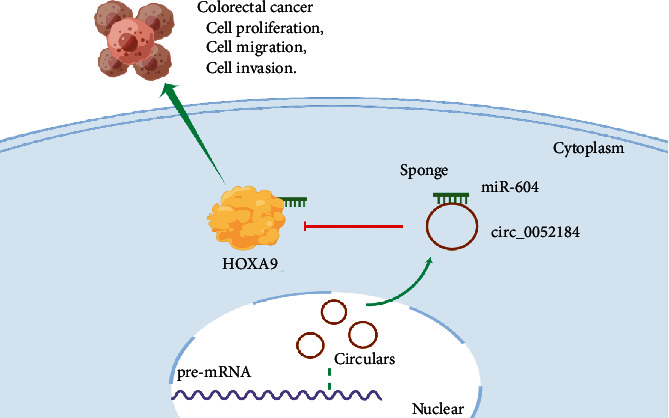
Schematic diagram: circ_0052184/miR-604/HOXA9 axis promotes the progression of colorectal cancer cell (by FigDraw).

Next, we further analyzed the downstream molecular mechanism of circ_0052184/miR-604. We predicted the downstream target genes of miR-604 through the online bioinformatics analysis website starBase (https://starbase.sysu.edu.cn/). The prediction results showed that there were 8 kinds of base pair complementation between miR-604 and HOXA9 (Figure 5(a)). The targeting relationship between HOXA9 and miR-604 was verified by constructing a dual-luciferase reporter assay system, cotransfected with HOXA9-MT or HOXA9-WT reporter systems with miR-604 mimics and negative controls; as shown in the dual-luciferase assay in Figure 5(b), the upregulation of miR-604 inhibited HOXA9-WT luciferase activity, but not HOXA9-MUT activity. Next, the expression of HOXA9 in CRC tissues and cells was detected by qRT-PCR, and the results showed that the expression of HOXA9 was increased in CRC tissues and cells (Figures 5(c) and 5(e)). The expression of the HOXA9 protein after miR-604 downregulation was analyzed by western blotting. And the results showed that when miR-604 was downregulated, the expression of HOXA9 increased (Figures 5(d) and 5(f)). Western blotting analysis showed that the HOXA9 protein level decreased when circ_0052184 was silenced, and the miR-604 inhibitor reversed the inhibitory effect of si-circ_0052184 on the HOXA9 protein level (Figure 5(g)). At the same time, Pearson correlation showed that the HOXA9 expression was inversely correlated with that of miR-604 (Figure 5(h)). In conclusion, there was a circ_0052184/miR-604/HOXA9 axis in colorectal cancer, which regulated the occurrence and development of colorectal cancer (Figure 6).

## 4. Discussions

Colorectal cancer is a common digestive tract cancer that threatens people's health [16]. At present, the morbidity and mortality of CRC patients are gradually increasing, and the 5-year survival rate is low with advanced CRC patients [17]. Therefore, exploring the molecular mechanism of CRC progression and clarifying the regulation of the process of advanced CRC progression will help to develop new clinical treatment strategies for advanced CRC patients. Dysregulation of circRNAs is related to CRC progression in existing studies, and circRNAs may regulate the development of CRC [18]. Our research explores the specific pathway that circ_0052184 promotes CRC progression.

Noncoding circRNAs are endogenous ncRNAs expressed in a variety of diseases and widely present in eukaryotic cells. circRNAs form covalently closed loops by back-splicing. This structure makes them highly stable and conserved [19]. The role of circRNAs in colorectal cancer has received increasing attention. A variety of circRNAs have been confirmed to be related to CRC development. circ_0014130 acted as a molecular sponge for miR-197-3p and participated in the drug sensitivity and development of colorectal cancer by regulating the miR-197-3p/PFKFB3 axis [20]. Downregulation of circ_0011385 in colorectal cancer inhibited cell proliferation and promoted apoptosis by regulating the miR-330-3p/MYO6 axis [21]. circ_0001666 can inhibit the progression of colorectal cancer by regulating the miR-576-5p/PCDH10 axis [22]. However, the effect of circ_0052184 on CRC was unknown. In our work, we verified that circ_0052184 was upregulated in colorectal cancer tissues and cells. We also determined the effect of circ_0052184 on colorectal cancer: silencing circ_0052184 in CRC cells inhibited the proliferation and migration and invasion of CRC cells *in vitro*. These revealed that the expression of circ_0052184 played a crucial key in regulating the occurrence and development of CRC cells. However, the regulatory mechanism of circ_0052184 remained further explored.

The knockdown or overexpression of circRNAs has an impact on the occurrence and development of cancer cells. There are various mechanisms for the regulation of circRNAs on tumors, such as miRNA sponges [23] and interaction with proteins [24]. One of the most common regulatory mechanisms is the circRNAs as endogenous competitive RNAs [25]. However, the role of circ_0052184 in colorectal cancer should be further explored. Firstly, we identified that the downstream target of circ_0052184 was miR-604 through circinteractome website prediction, dual-luciferase reporter gene experiments, and RIP experiments. The expression of miR-604 in CRC cells and tissues was reduced. Pearson correlation analysis showed that circ_0052184 expression and miR-604 expression were negatively correlated. Recent research results have shown that miR-604 is abnormally expressed in tumors such as nonsmall cell lung cancer [26], ovarian cancer [27], and gastric cancer [28]. Qiu et al. found that SNP predicted abnormal expression of miR-604 [29]. The results of colony formation assay, wound healing assay, and transwell assay indicated that silencing circ_0052184 inhibited the cell proliferation, migration, and invasion of colorectal cancer cells. Inhibiting the expression of miR-604 reversed this phenomenon. In conclusion, circ_0052184 regulated the occurrence and development of colorectal cancer by targeting miR-604. Our study revealed that miR-604 was a target gene of circ_0052184, and inhibition of miR-604 attenuated the development of colorectal cancer cells promoted by circ_0052184.

HOX genes are a subfamily of the homeobox gene family and are evolutionarily highly conserved [30]. Recent studies have confirmed that abnormal HOX gene expression induced malignant transformation, proliferation, and invasiveness of cells, leading to tumorigenesis [31]. HOXA9 gene was a cluster A HOX gene located on chromosome 7. Most studies have shown that the progression of multiple malignancies was associated with aberrant HOXA9 expression [32]. The HOXA9 mRNA level in colorectal cancer tissues and cells were detected by qRT-PCR, and the results were similar to the study by Bhatlekar et al.: HOXA9 is highly expressed in CRC, which revealed that HOXA9 promotes CRC progression [33]. We determined that there was a specific binding region between HOXA9 and miR-604 by starBase prediction and dual-luciferase reporter gene experiments. qRT-PCR, western blot analysis, and Pearson correlation indicated that HOXA9 interacted with the miR-604 expression. It was suggested that the miR-604/HOXA9 axis regulated the malignant development of CRC.

Taken together, our findings suggest that circ_0052184 promotes colorectal cancer progression by targeting the miR-604/HOXA9 axis (Figure 6). circ_0052184 is a newly discovered circRNA, and there were few reports on the regulatory effect of circ_0052184 on colorectal cancer. Exploring the regulatory mechanism of circ_0052184 might provide a theoretical basis for improving the clinical targeted therapy of colorectal cancer. At the same time, our study might provide a valuable reference for the mechanism of action of circ_0052184 in other tumors and also provide strong evidence for circ_0052184 as a clinical target for treatment. Of course, our study has some limitations, which need to be verified by vivo experiments. Other downstream target genes of circ_0052184 need to be screened by follow-up research.

## 5. Conclusion

The molecular mechanism of CRC is further refined by our work. This study confirmed that circ_0052184 regulated the miR-604/HOXA9 axis and revealed that the mechanism promotes the malignant development of CRC cells (Figure 6). Our work evidenced that circ_0052184 may be a therapeutic target for CRC and provided a theoretical basis for the exploration of CRC targeted therapy sites and biomarkers for CRC diagnosis.

## Figures and Tables

**Table 1:** Primers for circ_0052184, miR-604, and HOXA9.

Primers	Forward	Reverse
circ_0052184	5′-CGGTGGCAGCGGGAC-3′	5′-CCTCCAGGTGCTGGACT-3′
miR-604	5′-GCCGTATCCGCAATGTGTTAT-3′	5′-CGACGCTTGCGCGAG-3′
HOXA9	5′-CCCCGACTTCAGTCCTTGC-3′	5′-GATGCACGTAGGGGTGGTG-3′
U6	5′-CTCGCTTCGGCAGCACA-′3	5′-AACGCTTCACGAATTTGCGT-3′
GAPDH	5′-ACTCCTCCACCTTTGACGC-3′	5′-GCTGTAGCCAAATTCGTTGTC-3′

**Table 2:** Correlation of HOXA9 protein expression with clinical characteristics of CRC (*n* = 42).

		HOXA9 protein expression			
Category	Number	Low (*n* = 21)	High (*n* = 21)	Chi-square	*p* value
Sex				0.398	0.533
Male	18	8	10		
Female	24	13	11		
Age				0.0988	0.753
≥55	17	8	9		
<55	25	13	12		
Tumor size(cm)				5.08	0.024^∗^
<5	15	11	4		
≥5	27	10	17		
Lymph node metastasis				4.71	0.030^∗^
Negative	19	13	6		
Positive	23	8	15		
TNM stage				6.46	0.011^∗^
I+II	16	12	4		
III+IV	26	9	17		

## Data Availability

All data used to support the findings of this study are included within the article.
